# Efficacy and feasibility of the BREATHE asthma intervention with American Indian children: a randomized controlled trial

**DOI:** 10.1038/s41598-022-25447-0

**Published:** 2022-12-08

**Authors:** Rae A. O’Leary, Marcia A. O’Leary, Dara G. Torgerson, Raeann D. Mettler, Kendra J. Enright, Lyle G. Best

**Affiliations:** 1 Missouri Breaks Industries Research, Inc., 118 S Willow St, Eagle Butte, SD 57625 USA; 2 University of California, San Francisco, San Francisco, CA USA; 3 Turtle Mountain Community College, Belcourt, ND USA

**Keywords:** Asthma, Patient education, Paediatric research, Randomized controlled trials

## Abstract

American Indian (AI) children experience significant disparities in asthma prevalence, severity, and burden of disease, yet few asthma education interventions are tested in this population. This study aimed to evaluate the efficacy and feasibility of the BREATHE intervention with parents and AI children, during a 3-year follow-up period (n = 108), using a randomized controlled design. Children with asthma identified by electronic medical records (EMR) were screened and matched with 2 controls. The intervention included an initial educational and 24 months of follow-up. The control group continued their usual care. The primary outcome was the frequency of EMR documented, emergency department (ED) visits or hospitalization for respiratory complaints. There was no statistical difference in mean primary outcomes (1.34 (1.98) vs 1.22 (1.95), − 0.88 to 0.63, 95% CI of the difference, p = 0.75), nor percent with any ED visit or hospitalization (29/53, 55% vs 30/55, 54%, p = 0.99) between the intervention or control groups respectively. After 365 days, there was a borderline significant difference in time to primary outcome. Although limited in power, the present study did not demonstrate a persistent effect of this intervention. We recommend that AI pediatric asthma interventions are culturally-designed, use feasible procedures, and repeat education at least every 12 months.

## Introduction

Asthma is a disease that exposes disparities^1,2^. It is well documented that asthma diagnosis and poor control of asthma symptoms are associated with less education, poverty conditions, and minority status, specifically among American Indians (AI)/Alaska Natives (AN), possibly because of intersectionality of these socioeconomic risk factors^2,3^, genetic factors^4^, and immune factors^5^ in this special population. Racial and socioeconomic factors associated with increased asthma prevalence are commonly paired with reduced access to healthcare and increased environmental exposures, which can lead to poor asthma control and increased severity^1^. According to a 2019 report from the National Health Interview Survey, the prevalence of current asthma in AI/AN children was 14.6%, second only to Non-Hispanic Black children (16.4%)^6^.

A review from the Cochrane Library on culture-specific programs for children and adults from minority groups who have asthma found a total of seven randomized controlled trials (RCTs) with 837 participants from ethnic-minority groups who had asthma^7^. None of the qualifying studies included an AI/AN sample. Of the four culture-specific asthma education programs for children, favorable and significant changes were observed among the culture-specific intervention in the following outcome areas: (1) hospitalization for severe asthma exacerbation was reduced after 6 months in one study^8^ and after 12 months in another study^9^, (2) asthma control improved after 6 months in one study^10^, and after twelve months in another study^8^, and (3) asthma knowledge improved in three studies^8,10,11^. Based on the limited RCTs identified in this review, culture-specific asthma education programs are recommended, but more RCTs of feasible interventions are needed to strengthen the quality of evidence^7^. While the present study was not specifically culturally-adapted, it fills a critical need for more research on asthma education interventions feasible for AI/AN children with asthma in rural Tribal communities.

In 2007, an asthma education intervention, Breathing Relief Education and Tribal Health Empowerment (BREATHE) was developed and trialed by one of the authors. This pilot study enrolled 50 individuals with poorly controlled asthma living in a rural tribal community in the Northern Plains of South Dakota (mean age 32, range 6–62)^12^. Following a basic assessment and history intake at the baseline exam, the one-time 30-min asthma education intervention was delivered and customized to meet the participant's particular needs and situation. For example, time spent on particular asthma trigger avoidance (i.e. secondhand smoke or seasonal allergies) was adjusted based on relevance to the participant. Education reinforcements and case management occurred via phone at 1-month, 3-months, 6-months and 12-months. A review of medical records for a 12-month period pre-intervention and a 12-month period post-intervention revealed promising results. Participants experienced fewer Emergency Department (ED) and Urgent Care visits for asthma or other respiratory symptoms (15 pre-intervention; 6 post-intervention; *p* < 0.029); increased compliance with medications to control asthma (117 refills pre-intervention; 152 refills post-intervention) and a reduced need for systemic corticosteroids (19 pre-intervention; 13 post-intervention) (personal communication: Rae O’Leary).

In the present study, BREATHE was tested in a RCT as part of the Factors Influencing Pediatric Asthma (FIPA) study for a more rigorous examination of the intervention’s efficacy and feasibility. This study is important because asthma interventions can be costly and labor intensive and thus impractical in resource constrained rural Tribal communities, but this RCT trialed an efficient and cost-effective intervention for residual effectiveness following a one-time 30-min intervention. We hypothesized that AI children with asthma who received BREATHE would have fewer ED visits in the 3 years after receiving the intervention, compared with children randomized to receive their usual care. We also hypothesized that secondarily, BREATHE participants would have improved asthma control, knowledge, and quality of life, as well as reduced secondhand smoke exposure.

## Methods

### Trial design

This study aimed to evaluate the efficacy and feasibility of the 24-month long (range 15–35 months) BREATHE asthma intervention involving parental and child education, during a 3-year follow-up period for American Indian children (n = 108), using a randomized controlled study design. The duration of the BREATHE asthma intervention varied in duration due to funding deadlines. The ratio of intervention to control participants was 1:1 and there were no changes to the intervention or trial outcomes during the study period.

### Setting and participants

The FIPA Study was conducted among an American Indian population in a rural region in north-central United States, where tribal members primarily rely on Indian Health Service (IHS), and the tribal health department for healthcare. To receive specialty care for asthma from a pulmonologist or allergist, families travel 2–3 h. Challenges to access healthcare in this low income community, and suboptimal housing conditions contribute to excessive burden from asthma^2^.

The definitive diagnosis of asthma for both research and clinical purposes is difficult. We chose a case definition that is relatively conservative, practical in application, and more objective than patient/parent self-report of asthma. All participants met 2 of the 3 criteria for inclusion based on a chart review: (1) a diagnosis of asthma on at least 2 occasions by more than one provider during the past 2 years, (2) refills of asthma treatment medications on at least 2 occasions during the past 2 years, or (3) improvement in forced expired volume at one second (FEV1) of at least 20% with a short-acting bronchodilator. Exclusionary criteria were (1) neonatal ventilator treatment, (2) hospitalization at birth lasting more than 15 days, (3) congenital heart anomaly requiring surgery, (4) diagnosis of cystic fibrosis, (5) congenital lung, diaphragm, chest wall, or airway anomaly, (6) diagnosis of pneumonia, pertussis, or tuberculosis within the past year, or (7) congenital muscular disorder.

Cases were randomly assigned to either the BREATHE arm described above, or to the control Non-Intervention arm, consisting only of the child's usual and customary clinical care and a packet of written material about asthma management provided by study staff at enrollment. After consent and assent was obtained by research staff, participants were randomized without replacement by allowing the participant to blindly draw one of two different colored balls from a container for an equal chance to be in either group. The research staff recorded the color chosen and the participant signed an acknowledgement of the color. A total of 108 participants with asthma were enrolled; 53 were randomly selected to receive BREATHE, 53 were randomly selected to be in the Non-Intervention group, and 2 did not consent to participate in the RCT portion of the study so they were assigned to the Non-Intervention group. Staff were aware of participants’ assignment to intervention or non-intervention arms. Additional details on setting and participants were previously described^2,4,5^.

The research protocol was approved by the Institutional Review Boards at the Collaborative Research Center for American Indian Health, Sanford Research, Sioux Falls, SD; the Great Plains Indian Health Service area office in Aberdeen, SD, and the local tribal government. All research was performed in accordance with relevant guidelines/regulations. Participants’ parents gave informed consent in writing and children provided assent. The study was not preregistered, but it was registered on 05/10/2017 with the US National Library of Medicine on clinicaltrials.gov as the “Factors Influencing Pediatric Asthma Study, identifier NCT03302962.

### Recruitment

Children with asthma were identified through an automated query of the IHS and tribal health department electronic health records using the International Classification of Diseases, ninth edition (ICD-9) code 493.9 within the ages of 6 and 17 years. Additional potential participants were identified through local providers at other healthcare facilities. These recruitment methods resulted in over 700 children who were identified as potentially meeting inclusion criteria, of which about 450 were excluded because they lacked a clinical provider diagnosis of asthma (rather than a pharmacy diagnosis automatically assigned to those with a bronchodilator prescription), or lacked repeated asthma medication refills. Approximately 130 of the remaining 250 were able to be contacted—of those, some declined the medical record review to determine eligibility, and others consented to the medical record review, but were ineligible, leaving 108 cases interested and eligible to participate in the study.

Because this study used a convenience sample in a small community with a limited number of children that would meet inclusion criteria, no sample size or power calculations were used.

### Measures

The primary outcome measure was the number of Indian Health Service ED visits for respiratory complaints during the observation period from 9/1/13 through 5/25/17. The electronic medical records, including dates of visit, for all participants were available for the entire observation period, thus there was no "loss to follow-up" for our primary outcome, although it is recognized that some participants may have accessed other facilities, resulting in records that were unavailable to us. This was analyzed both as a mean number of visits per participant or dichotomized as none vs ≥ 1.

Secondary outcomes were measured following consent and randomization procedures using a self-administered questionnaire completed by the participant and a guardian depending on the child’s age at the initial exam and final exam. Using the Check Your Asthma I.Q. Quiz^13^, we assessed current asthma knowledge of the child if age 10–17, or the parent if the child was age 5–9, in which each point represents the number of correct responses out of 12 points. Severity of disease was assessed using the Childhood Asthma Control Test™^14^, for parents and children ages 5–11 (27 points possible; scores ≥ 20 indicate well controlled asthma), or the Asthma Control Test™^14^, for children ages 12–18 (25 points possible; scores ≥ 20 indicate well controlled asthma). Present quality of life was assessed using the Asthma Life Quality Test^15^, in which each point represents the number of quality of life items impacted by asthma out of 20 possible points, so a greater score indicates worse quality of life. Parents and children of all ages responded to the Asthma Life Quality Test independently, but in the presence of each other with the child answering before the parent.

A saliva Accutest NicAlert™ (Jant Pharmacal Corp, Encino, CA) assay was used to assess salivary levels of cotinine; categorical scores range from 0 which identifies a non-tobacco user, to scores of 1, 2, 3, 4, 5, or 6 which identifies tobacco users or individuals highly exposed to environmental tobacco smoke (ETS) to varying degrees^16^.

### Treatment conditions

Following the baseline questionnaire and exam, participants randomized to the BREATHE arm received comprehensive information from a single asthma counselor about asthma and the management of the disease was discussed with caregivers involved in the care of the participant. Materials from the American Lung Association’s “Controlling Asthma: What You Need to Know” flipbook was used for education^17^. The asthma counselor emphasized medication use and compliance, a customized asthma action plan, and the deleterious effects of smoking. The asthma counselor had no previous experience delivering asthma education, but was trained by an experienced certified asthma educator to deliver the intervention and had a 4-year medical degree. Control participants were given an educational packet and encouraged to maintain regular contact with their usual provider; no case management calls or questionnaires were attempted between baseline and final exam.

After the baseline education, standardized case management procedures were conducted by research staff. Two to four weeks after education, contact was attempted via phone to repeat the questionnaire, discuss questions or concerns, and coordinate care if needed. Case management was attempted every 3 months for the first year, every 6 months during the second year, for a total of 6 possible case management sessions between the baseline and final exam, which ranged from 15 to 35 months. Content and questionnaires were the same for all educational and case management sessions. Initial exams that included the BREATHE Intervention or the control conditions began in September 2013 and were concluded in March 2015. Final exams occurred 13 to 33 months after the baseline exam, from January 2016 to December 2016.

### Statistical analysis

Kaplan–Meier analysis was undertaken to analyze our primary outcome of the differences in the number of days to first ED visit or hospitalization for respiratory problems between the BREATHE arm and the Non-Intervention control arm. The "time to event" was calculated from the participant's enrollment exam (synchronous with the start of intervention) and ended with the defined endpoints of first ED visit or hospitalization, as noted above. We also compared the rate of ED visits or hospitalization over the entire study period using Poisson regression. Analysis for secondary outcomes included a *t*-test for independent samples to test for group mean differences in quantitative variables, whereas chi-square tests were used to evaluate differences in discrete variables. A paired *t*-test or a McNemar's chi-square test was used to compare an individuals' change from baseline to final contact. Quantitative comparisons were two-tailed. All data analysis was pre-specified.

Data was entered into Microsoft Excel, and STATA 14.2 (Stata Corp, College Station, TX, USA) was used to identify outliers to be verified or corrected. All other analyses were conducted on SPSS 13.0 (SPSS Inc., Chicago, IL, USA) software.

## Results

**Figure 1:**
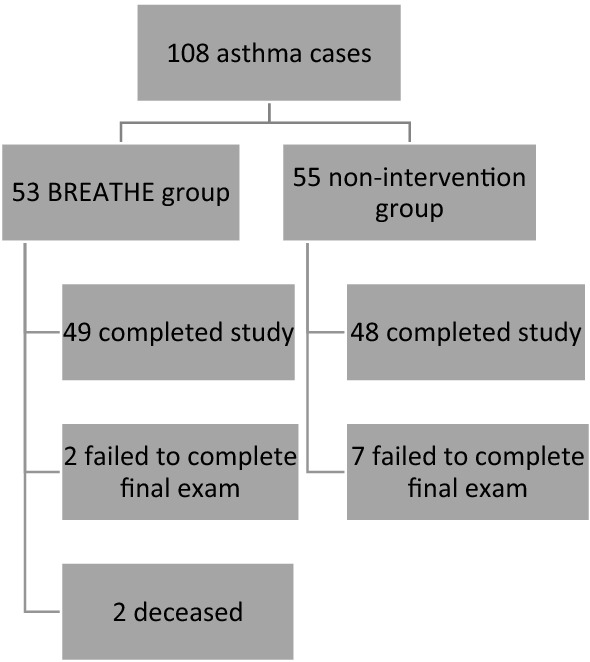
Consort diagram.

There was a total of 108 children with asthma who enrolled in the FIPA Study (Fig. 1). Nearly half were randomized to BREATHE (*N* = 53) and the others were assigned to the Non-Intervention arm (*N* = 55). Two participants did not consent to receive BREATHE so they were placed in the Non-Intervention group.

The primary outcome of interest was an ED visit and/or hospitalization recorded in the IHS electronic medical record for respiratory problems among the 108 RCT participants. During the 3-year observation period there were a total of 29 events for the BREATHE arm and 30 for the Non-Intervention arm. In a comparison of ED visits and hospitalizations following study enrollment (9/1/13 through 5/25/17), comparing the BREATHE group with controls, neither mean ED visits and hospitalizations (1.34 (SD = 1.98) vs 1.22 (SD = 1.95), -0.88 to 0.63 is the 95% CI of the difference and p = 0.75), nor percent of participants with any ED visit or hospitalization (29/53, 55% vs 30/55, 54%, p = 0.99) differed respectively by group. No harms were identified by the data safety management board (DSMB) for either group, so the trial was carried out through full the intended timeframe and ended due to the completion of funding.

**Figure 2:**
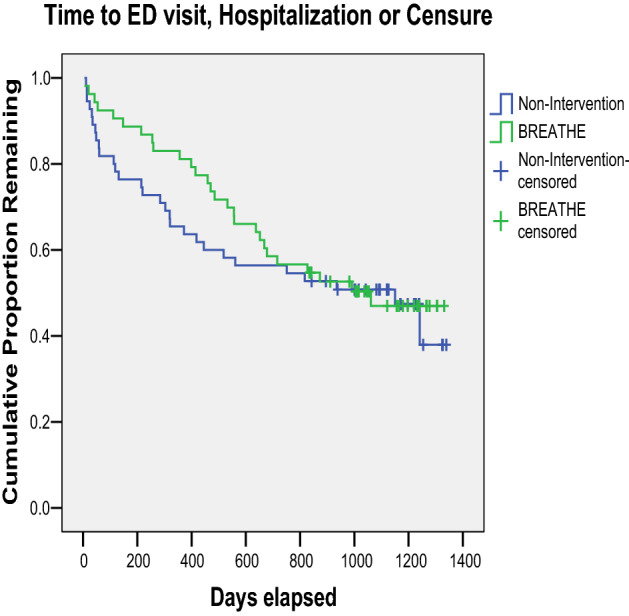
Time to event from participant intervention initiation to first ED visit or hospitalization, by group status.

Kaplan–Meier time-to-event analysis is shown in Fig. 2. The mean days to endpoint (and 95% confidence intervals) for BREATHE and Non-Intervention were 881 (752–1011) and 796 (649–943), respectively, which clearly overlap. The log rank test (Mantel-Cox) indicated a chi-square of 0.271 and *p*-value of 0.603. Although the planned evaluation period was within the time available, which was a maximum of 1362 days, if alternate endpoints are chosen, at 730 days from the exam date, the log rank chi square test = 0.215, *p* = 0.643. If restricted to the first 365 days post exam, the results approach statistical significance, log rank chi square = 3.420, *p* = 0.064.

The overall rate of ED visits and hospitalizations throughout the duration of study follow-up using Poisson regression did not show any significant difference between individuals who received the BREATHE intervention and those who did not (p = 0.83).

Of the 53 participants that received BREATHE, 49 completed the final exam to assess secondary outcomes (92.5% retention rate), 2 did not complete the final exam and 2 were deceased (causes of death were unrelated to asthma or other respiratory illnesses). Analysis of the number of case management calls for the 49 BREATHE participants that completed the final exam was an average of 32% of calls completed in the first year, and 26% of calls completed in the second year. Out of the 6 possible case management calls in the first and second year combined, call completion ranged from 0 to 4, with a median 2, mode 2, and a mean 1.77 (SD = 0.93). Of the 55 Non-Intervention participants, 48 of 55 completed the final exam (87.3% retention rate).

Overall, no significant differences were found between randomized study arms, with the exception of one baseline demographic (Table 1). There were proportionally fewer married parents in BREATHE compared to the Non-Intervention arm (18.9% vs. 38.2% *p* = 0.027). Measures of interest at baseline in Table 2 also had one variable, smoking allowed outside of home, that differed by group status (58.5% vs 78.2% *p* = 0.028).

**Table 1:** Baseline demographic, socioeconomic, and household environmental characteristics by group status.

	BREATHE intervention (*N* = 53)		Non-intervention (*N* = 55)	
	Mean (SD)	N (%)	Mean (SD)	N (%)
**Demographic characteristics**				
Age at first exam	12.24 (3.39)		11.39 (3.02)	
Male gender		23 (43.4)		34 (61.8)
**Socioeconomic characteristics**				
Currently married		21 (18.9)		10 (38.2)
Years of education	12.59 (1.95)		12.56 (2.59)	
Less than high school education		11 (20.8)		8 (14.5)
High school education		24 (45.3)		33 (60.0)
Beyond high school education		16 (30.2)		14 (25.5)
Income less than 25,000		33 (62.3)		43 (78.2)
Government health insurance		44 (83.0)		49 (89.1)
Home ownership		17 (32.1)		15 (27.3)
**Household environmental characteristics**				
Single unit home		41 (77.4)		36 (65.5)
Home occupants, 4 or less		25 (47.2)		21 (38.2)
Home occupants, 5 to 8		21 (39.6)		31 (56.4)
Home occupants, 9 or more		5 (9.4)		3 (5.5)
Reported rodent or insect infestation		3 (5.7)		4 (7.3)
Reported water damage in the home		13 (24.5)		10 (18.2)
Use of wood burning stove		3 (5.7)		3 (5.5)
Pets in the home		37 (69.8)		37 (67.3)
Reported tobacco smoke exposure		38 (71.7)		45 (81.8)
Report of home environment worsening child's health		30 (60.6)		33 (61.1)

**Table 2:** Baseline outcomes of interest by group status.

	BREATHE intervention (*N* = 53)			Non-intervention (N = 55)		
**Lab test**	**N (%)**			**N (%)**		
NicAlert positive for cotinine	38 (71.7)			40 (72.7)		
**Questionnaire (self-report)**	**N (%)**			**N (%)**		
Persistent cough in past 4 weeks	23 (46.0)			28 (50.9)		
Wheezing in past 4 weeks	24 (48.0)			32 (59.3)		
Difficult time breathing in past 4 weeks	21 (42.0)			31 (56.4)		
Prescribed meds not taken	11 (21.6)			13 (23.6)		
Smoking allowed in home	17 (32.1)			11 (20.0)		
Smoking allowed outside of home	31 (58.5)			43 (78.2)		
Smoking allowed in vehicle	17 (32.1)			21 (38.2)		
**Questionnaire (self-report)**	**Mean (SD)**			**Mean (SD)**		
Asthma Control	18.57 (4.09)			19.69 (4.38)		
Asthma life quality—child	9.24 (4.22)			7.88 (4.36)		
Asthma life quality—parent	10.27 (4.54)			9.04 (4.74)		
Asthma knowledge	8.90 (1.64)			9.02 (1.62)		

Chart review (24 months prior to enrollment)	Mean (SD)	Median (interquartile range)	Minimum/maximum	Mean (SD)	Median (interquartile range)	Minimum/maximum
Rescue med refills	3.28 (3.90)	2 (0–5)	0/17	4.00 (3.86)	3 (1–6)	0/19
Control med refills	2.80 (4.92)	1 (0–3.25)	0/27	3.71 (6.82)	1 (0–4)	0/32
Steroids	0.67 (1.16)	0 (0–1)	0/5	1.04 (1.18)	1 (0–2)	0/5
Hospitalizations	2 among 2 participants			2 among 2 participants		
General clinic visits	2.67 (4.80)	1 (0–3)	0/32	2.00 (2.06)	1.5 (0–3)	0/8
Total ED visits	1.18 (1.88)	1 (0–1)	0/8	1.08 (1.66)	0.5 (0–2)	0/9
Participants with any ED visit	26/49(53%)			26/52(50%)		

In addition to the primary outcomes previously described, Table 3 shows between group comparisons of change in secondary outcomes. In both groups, NicAlert scores increased, indicating more tobacco use or exposure to ETS; the BREATHE arm mean change − 0.36 (SD = 1.24) and the Non-Intervention arm mean change − 0.65 (SD = 0.15), *p* = 0.31. Mean change in Asthma Knowledge − 3.33 (SD = 1.72) in the BREATHE arm and − 3.79 (SD = 2.13), *p* = 0.27 in the non-intervention group indicate lower scores over time. No mean changes were statistically significant.

**Table 3:** Baseline versus final outcomes, between group change.

Individual change between groups, mean (SD)^a^				
	Mean change BREATHE	Mean change Non-intervention	95% CI of difference	p-value
NicAlert change^b^	− 0.36 (1.24)	− 0.65 (0.15)	− 0.85 to 0.27	0.31
Asthma control change	0.40 (5.52)	0.27 (4.87)	− 2.33 to 2.08	0.91
Asthma life quality—child change	1.45 (4.89)	1.45 (5.54)	− 2.28 to 2.27	1.00
Asthma life quality—parent change	1.87 (5.49)	1.51 (5.98)	− 2.16 to 2.88	0.78
Asthma Knowledge	− 3.33 (1.72)	− 3.79 (2.13)	− 1.28 to 0.36	0.27

Between group comparisons of pertinent outcome measures				
	BREATHE^c^	Non-intervention	95% CI of difference	*p*-value
ED visits and hospitalizations, mean (SD)^d^	1.34 (1.98)	1.22 (1.95)	− 0.88 to 0.63	0.75
Participants with any ED visits or hospitalizations, yes/total (%)	29/53 (55)	30/55 (54)		0.99

^a^Positive values indicate improvement, note: to account for differences in labeling:

Nicalert change = baseline − final (less nicotine exposure), and Asthma Life Quality change = baseline − final (increased quality of life); but asthma control change = final − baseline (improving control), asthma knowledge change = final – baseline.

^b^Categorical variable with 6 possible, higher values = increased exposure.

^c^Mean (SD) for continuous variables, count/total (%) for discrete variables.

^d^9/1/13 through 5/25/17.

## Discussion

While the BREATHE Asthma Intervention piloted in 2007 showed promising results to reduce asthma or respiratory-related ED visits (personal communication: Rae O’Leary), the present RCT of the BREATHE Intervention trialed in the same community with a pediatric population was not confirmatory. When comparing the BREATHE arm and Non-Intervention arm in the survival curve of time to ED visit, the groups appeared to diverge early in the study. However, the groups converged around day 600, with no significant difference overall. It is possible that an initial impact was made during the first 12-months when proximity to the asthma education session was more recent, but this impact was not sustained over time with sporadic phone contacts throughout the course of the 24 month intervention period. Other possible contributors to the lack of effect and lessons learned to support future asthma interventions for AI/AN youth are discussed below.

There were a number of differences between BREATHE from the pilot study in 2007 to the present study, as well as challenges faced with the implementation of the intervention protocol.

First, the qualifications of asthma counselors varied from the pilot to the present study which may have impacted the fidelity of the intervention. The American Indian asthma counselor delivering the intervention during the pilot was a certified asthma educator and had prior experience in asthma education, whereas the American Indian RCT interventionist had formal medical experience and education, but was not a certified asthma educator; both had 4-year health-related degrees. In the pilot, educational reinforcement regarding medications and incorrect responses on the asthma knowledge questions was provided, but this was not standard protocol during case management calls for the RCT.

Secondly, there was limited engagement with case management phone calls that were attempted every 3 months for the first year, and every 6 months during the second year. Many calls were missed entirely due to changed contact information, or interfering schedules. Sometimes families were able to be reached but the phone call was conducted with variable individuals (child participant, consenting parent, or occasionally another caregiver). It is also possible that participating families did not answer calls because they felt the time commitment was too great, or they were simply not interested. A monetary compensation was offered for the participant’s time for the baseline and final exam, but not for the phone contacts. This may have also been a contributing factor to the limited case management calls. It is conceivable that if a consistent person from the participating family was able to be contacted for every phone call by a certified asthma educator, that ED visits would be reduced using BREATHE. The challenges faced may indicate that the intervention protocol is not feasible without an incentive for case management calls and may need modified to be deliverable by an individual without relevant experience or certifications.

Third, the BREATHE pilot was tested with all ages, not just a pediatric sample. In the pilot, because participants were predominantly adults, the case management calls were completed consistently with the participant who conceivably had greater interest in improving asthma management since they were directly impacted by poor disease management. Whereas, in the RCT, the parent or caregiver completing case management calls was variable and not directly impacted by asthma symptoms, and therefor has less motivation to engage.

Finally, participants in the BREATHE pilot had worse asthma control (based on ACT score) at baseline due to inclusion criteria, but participants with well-controlled asthma took part in the RCT. Again, the greater potential for well-controlled asthma in the RCT may have served as a deterrent to engagement in the BREATHE intervention.

It is likely that the limited case management calls, fidelity of the implementation of BREATHE and possibly reduced motivation among parents or caregivers negatively affected asthma knowledge scores from baseline to final. As a result, future iterations of this intervention may produce a reduction in ED visits if participants have poorly controlled asthma at baseline, face-to-face intensive asthma education is repeated 12 months after the baseline exam, and a more feasible educational protocol is implemented.

Despite the lack of impact on the primary outcome, ED visits, the pursuit for an effective, feasible, and culturally-designed pediatric asthma intervention must continue. The authors feel that of the few culturally-tailored asthma interventions we identified in our review of the Cochrane Library^8–11^, none were delivered in a way that is feasible and sustainable in a rural Tribal setting with limited medical resources. For example, the culturally-adapted CALMA asthma intervention for Puerto Rican children with asthma published by Canino et al*.* requires two in-home visits^8^, which would be difficult in remote Tribal communities and may not be culturally accepted, or the culturally contextualized “Healthy Breathing” asthma education program for Indian children with asthma published by Grover et al*.* has a more feasible structure, but is delivered by a pharmacist^10^, which is not reasonable in Tribal communities with professional healthcare worker shortages.

The use of a randomized, controlled trial provides stronger evidence of efficacy if significant results are obtained. Contrarily, a type II error will occur if the null hypothesis is accepted, but the study is under powered to detect an effect size of importance. The BREATHE pilot study reduced the historical incidence of mean ED visits by approximately 60% and if that effect was obtained in the present study, the current study would have required 111 participants in each arm to achieve a power of 80% with an alpha of 0.05^18^. Unfortunately, the ability to recruit from our available population was limited and the baseline prevalence of ED visits among pediatric asthma patients was not known with any precision. Our reported results involve multiple statistical tests, but we have continued to use a nominal p value threshold of 0.05. Although ideally, the staff person collecting the secondary measures would be a different person than the asthma counselor, in the present study, they were one and the same. We feel this was unlikely to seriously bias the measures collected, however, since some (eg NicAlert) were objective and many of the others were derived from self-administered questionnaires. Despite these limitations and the lack of effect, this study begins to fill the research gap about asthma education interventions for AI/AN children with asthma.

## Conclusions

While this study was limited in power and implementation feasibility, the BREATHE intervention did not have the intended effect of a sustained reduction in respiratory-related ED visits and hospitalizations following the one-time 30-min intervention. However, the intervention resulted in a borderline significant difference in time to primary outcome for 365 days post-intervention and contributed valuable lessons learned. We recommend the following considerations for pediatric asthma interventions designed for rural AI/AN populations.

1. Design education materials to be consistent with the community’s culture and social norms.
2. Prioritize in-person case management over phone calls, or incorporate sessions in conjunction with other settings where the participant is already going, such as clinic appointments, or school to maximize the convenience for participating families.
3. Determine a feasible frequency and method for ongoing case management. Brief sessions every 6 months and as needed may improve efficacy. Another consideration is to use an app- or computer-based program for participants to respond.
4. Implement educational algorithms that can be used by a lay-person or an electronic program, to prompt consultation with a trained asthma educator or healthcare professional only if indicated.
5. Repeat intensive in-person education at least every 12 months.

## Data Availability

Rae O’Leary may be contacted about the data, however, datasets used and/or analyzed during the current study are not publicly available due to tribal policy.

## Abbreviations

AI: American Indian
AN: Alaska Native
BREATHE: Breathing Relief Education and Tribal Health Empowerment
ED: Emergency department
ETS: Environmental tobacco smoke
FEV1: Forced expired volume at one second
IHS: Indian Health Service
RCT: Randomized controlled trial
